# OmicsVis: an interactive tool for visually analyzing metabolomics data

**DOI:** 10.1186/1471-2105-13-S8-S6

**Published:** 2012-05-18

**Authors:** Philip Livengood, Ross Maciejewski, Wei Chen, David S Ebert

**Affiliations:** 1 Department of Electrical and Computer Engineering, Purdue University, West Lafayette, IN, USA; 2 School of Computing, Informatics and Decision Systems Engineering, Arizona State University, Tempe, AZ, USA; 3 State Key Lab of CAD & CG, Zhejiang University, China

## Abstract

When analyzing metabolomics data, cancer care researchers are searching for differences between known healthy samples and unhealthy samples. By analyzing and understanding these differences, researchers hope to identify cancer biomarkers. Due to the size and complexity of the data produced, however, analysis can still be very slow and time consuming. This is further complicated by the fact that datasets obtained will exhibit incidental differences in intensity and retention time, not related to actual chemical differences in the samples being evaluated. Additionally, automated tools to correct these errors do not always produce reliable results. This work presents a new analytics system that enables interactive comparative visualization and analytics of metabolomics data obtained by two-dimensional gas chromatography-mass spectrometry (GC × GC-MS). The key features of this system are the ability to produce visualizations of multiple GC × GC-MS data sets, and to explore those data sets interactively, allowing a user to discover differences and features in real time. The system provides statistical support in the form of difference, standard deviation, and kernel density estimation calculations to aid users in identifying meaningful differences between samples. These are combined with novel transfer functions and multiform, linked visualizations in order to provide researchers with a powerful new tool for GC × GC-MS exploration and bio-marker discovery.

## Introduction

In recent years, GCxGC-MS has become an invaluable laboratory analysis tool. However, this procedure produces large (gigabytes of data per sample), four dimensional datasets (retention time one, retention time two, mass and intensity). Such data is cumbersome, and researchers must spend time formatting and processing the data in order to remove acquisition artifacts, and quantify and identify chemical compounds [1]. Furthermore, while statistical analysis has played an important role in this work (because of the need to reduce the thousands of acquired spectral features to a more manageable size), the large data size, inherent biological variability and measurement noise makes the identification of bio-markers through purely statistical processes extremely difficult and time consuming.

This work presents a visual analytics environment for analysis and visualization of metabolomic data obtained by GCxGC-MS. The system is in the form of a comparative visualization tool kit that allows users to quickly perform comparisons across and between sample sets, in order to enable advanced analytic exploration of GCxGC-MS data sets. The guiding principle is to utilize the ability of the visual perception system to cluster, detect outliers, and find patterns much more efficiently than the most advanced computerized algorithms. By balancing the statistical analysis with advanced visualization techniques in order to analyze and understand differences between samples, OMIC researchers are able to target analysis and re ne algorithms in order to begin to identify cancer biomarkers. These biomarkers are the key to identifying differences in susceptibility and treatment response for patient groups.

In this paper, we describe a new, efficient visual analytics suite of tools for GC × GC-MS data sets to be used in metabolomics for a variety of applications including cancer signatures detection. Our work is being developed in collaboration with analytical chemists and biology researchers, and is designed to provide an interactive, integrated visual and statistical analysis environment with multiple functionalities. Compared to our previous work [2] that processes a GC × GC-TOF dataset as a 2D (TIC) image, our expanded system presents and handles mass spectral data as three-dimensional distribution data. We employ volume visualization to depict and compare spectral distributions in a portion or a set of data, showing the individual spectral distribution for each pair of retention times, instead of the integrated sum. We perform comparative visualization on selected regions and potential bio-markers for identifying distinctive metabolic differences among a set of samples. System features include:

1. A comparative visualization system that allows multiple samples (and multiple views of individual samples) to be displayed and explored simultaneously,

2. Data exploration tools for exploring mass spectra and filtering and comparing TIC images in real-time,

3. Grouping and rendering of samples and calculation of group means for comparison and difference calculation,

4. Comparative volume rendering across samples.

### Related work

In order to develop a system that is both effective and efficient at finding differences across samples, this system builds on previous work from several areas. In particular, previous work from GCxGC-MS analysis and data visualization were examined. With regards to visualization, areas of relevance include focusing and linking techniques, comparative visualization, multi-dimensional visualization, user interface techniques and design, and the use of color as it relates to data and difference visualization.

### Two-dimensional gas chromatography-mass spectrometry

A traditional gas chromatograph consists of a carrier gas, injector port, column, detector and a recorder. The injector is set to be hotter than the boiling point of the sample so that the sample will be vaporized. The carrier gas flows through the system and pushes the gaseous components of the sample into the column. Generally, an inert gas, such as helium, is used. As the carrier/sample mixture moves through the column, the different components of the sample interact with the column and these interactions cause the samples to pass through the column at different rates. This chemical separation is used to classify the compounds within the sample, as each compound will exhibit a characteristic retention time. A detector resides at the end of the column, quantizing the output. The most common type of detector is a mass spectrometer that ionizes the gas as it elutes from the column, producing additional separation in the mass spectral domain [3]. This type of setup is referred to as Gas Chromatography-Mass Spectrometry, or GC-MS. GC-MS has proven to be very effective for simple mixtures. However, as the complexity of the samples increases, it becomes difficult to achieve adequate chemical separation. Different components will exit the column at the same or nearly the same time, a problem known as coelution. This results in partial or full overlap of peaks in the mass spectral domain. To help overcome this problem, two-dimensional gas chromatography-mass spectrometry (GCxGC-MS) has become a common chemical analysis tool.

There are two columns in the two-dimensional gas chromatography-mass spectrometry. Rather than going to a detector, the output of the first column goes into a modulator that collects the sample and periodically injects it into a second column with different chemical properties. Due to the different properties of this second column, compounds will have a different retention time in the second column, resulting in two levels of chemical separation. The detector is positioned at the output of the second column. This process results in a four-dimensional dataset with two time axes (retention time one (RT1) from the first column and retention time two (RT2) from the second column), mass, and intensity. For each RT1/RT2 coordinate pair, the mass and intensity form a mass spectrum.

An understanding of the structure, strengths, and shortcomings of GCxGC-MS data is essential in order to effectively apply visualization techniques. GCxGC-MS is known for its large peak capacity, an order of magnitude increase in chemical separation ability compared to GC-MS [4], and its improved speed [3]. However, GCxGC-MS still has several complexities that make it a challenging process to use effectively. Inconsistencies in sample amounts will lead to differences in peak intensities. Peak retention times and shapes experience slight differences that are uncontrollable, but are not related to actual chemical differences in the samples [4]. Data samples exhibit background noise that can vary from sample to sample, and make accurate peak quantification difficult. Finally, despite the improved separation ability over one-dimensional GC-MS, GCxGC-MS still exhibits some peak overlap due to coelution.

Algorithms that attempt to correct these deficiencies have been developed with promising results, but this is still an active area of research. To correct for differences in sample amounts, normalization can be performed. As GCxGC intensities are fairly linear with respect to sample amounts, a multiplicative scaling factor can be used to normalize samples as long as at least one consistent peak can be found to normalize against [4]. While fairly straightforward, background levels and peak co-elution can hinder the accuracy.

Several peak alignment techniques have been developed to correct the problem of peak shift in chromatography, such as the piecewise automated beam search algorithm developed by Yao et al. [5], or the registration technique described by Hollingsworth et al. [4]. Reichenbach et al. [6] found that the background level was mainly the sum of two slowly changing functions: a steady-state standing-current offset and a temperature-induced column bleed. They found that this background level varies slowly compared to the characteristic peak widths, and has the statistical characteristics of random white noise. With this knowledge they were able to develop an efficient algorithm for removing the background from a total ion count image, resulting in a near-zero mean background level.

Very often one would like to identify, or search for, a particular analyte of interest within a GCxGC-MS sample. These identification algorithms generally involve and correlation with samples from a known mass spectral library, such as in [7]. These algorithms, however, are complicated by coelution. In order to remove the overlap in the mass spectral domain that results when multiple components elute at the same time, spectral peak deconvolution algorithms have been developed, such as [8]. In general, however, these algorithms are dependent on there being at least some separation between the centers of the overlapping peaks. Due to such issues, the visual inspection and explorations of samples plays a vital role in the analysis process.

### GCxGC-MS visualization techniques

Several visualization techniques have been developed for exploring GCxGC-MS data and are used almost universally. Typical methods include: two-dimensional total ion count rendering [3,4], two-dimensional contour plots, three-dimensional height rendering, or displaying the data in tabular form [3]. Additionally, subsets of the data can be displayed, such as mass-spectrum views for a given retention time coordinate pair, or visualizing a single mass or range of masses using any of the previously mentioned methods. The scientific community has recently begun taking advantage of modern graphics hardware for more detailed, complex and realistic visualizations for all sorts of complex datasets, and mass spectrometry is no exception. Corral et al. [9] develop some basic hardware-accelerated rendering techniques for very large Liquid Chromatography-Mass Spectrometry (LCMS) datasets, adapting terrain-rendering techniques to suit the LCMS data. This height rendering technique is also used by Linsen et al. for differential protein expression analysis [10]. Their work also includes data resampling, as well interactive modification of the color scheme and material properties. Other research combines mathematical and statistical methods with visualization in order to provide more insight into data. Recent work by Wiklund et al. [11] makes use of an orthogonal partial least-square (OPLS) model-based approach for definition of statistically and potentially biochemically significant compounds. Wiklund et al. present the S-plot, visualizing both the covariance and correlation between metabolites and class designation, and an extension dubbed the SUS-plot (shared and unique structure) to compare the outcome of multiple classification models compared to a common reference.

However, little research has gone into interactive comparative visualization techniques for this type of data. Hollingsworth et al. utilized image processing based techniques based on the total ion count image [4]. This basically amounts to a flattening of the data from four dimensions to three by aggregating the individual intensities at each retention time coordinate in order to obtain the total ion count. Prior to visualization and comparison each image must undergo background removal and peak detection. Peak alignment is performed on the reference image in order to remove incidental differences with the analyzed image. Finally, normalization is performed so that differences in sample amounts will not result in false positives. Once this pre-processing is complete, a difference image is computed. This difference can be visualized using tabular data, a 2D image, or 3D height field visualization in either grayscale or color. Their main contribution, however, is the calculation of a fuzzy difference that compares each pixel value in one image with a small neighborhood of pixels in the other, rather than doing a pixel-by-pixel comparison. This technique helps to reduce the incidental differences in peak shape and retention time that may still exist even after alignment. While these techniques represent a good first step, they are ill-suited for biomarker detection as they rely solely on images produced from the total ion count image. Additionally, since only the magnitude of the difference is considered, the results obtained can be misleading. Large, yet statistically insignificant differences may be emphasized while small, yet significant differences may not be noticeable.

### Visual analysis system

We present and handle mass spectral data as both two and three-dimensional distributions through the use of novel interactive techniques. We allow users to explore their data using coordinated multiple views as shown in our previous work [2]. Furthermore, we have also expanded these tools to include dynamic filtering and selection, as well as three dimensional volume rendering of the data. In this section, we illustrate various components of our visual analysis system.

### Total Ion Count (TIC) visualization

The first component that we show is the TIC visualization. The total ion count is a reduction of the data set from four dimensions down to three. For each retention time coordinate, the intensities of the entire mass spectrum at that point are summed together to obtain the total intensity, or total ion count. Once the TIC data has been computed, it can be visualized either in 2D, or as a 3D height rendering.

#### 2D TIC

**Figure 1:**
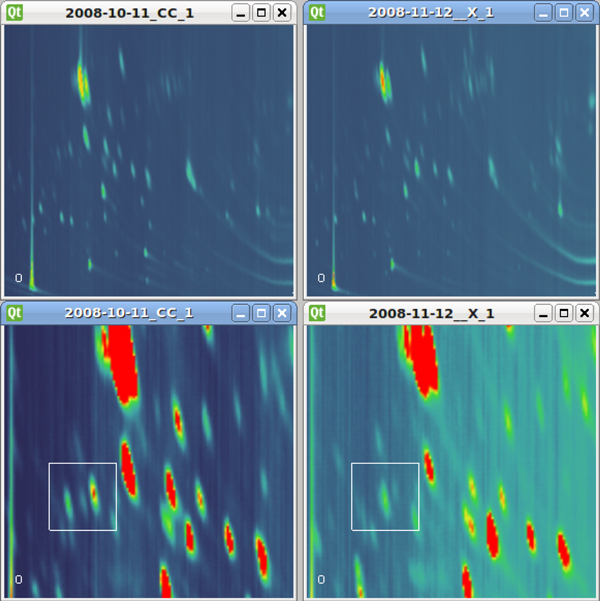
**GCxGC-MS data sets**. GCxGC-MS data sets rendered as 2D TIC images (top). Our system provides visual analytic tools to help researchers identify meaningful differences (bottom). Here we show a canine cancer sample (left) compared with a healthy canine sample (right).

The two-dimensional total ion count image is one of the most common visualization techniques for GCxGC-MS data. Ion count values are mapped to a color using the specified transfer function and rendered to the screen as a flat, two-dimensional image, as in Figure 1.

#### 3D TIC

A total ion count height rendering is nearly identical to the two-dimensional total ion count image. For the height rendering, the intensity is used as the z-coordinate in a polygonal mesh, and can be scaled linearly or logarithmically to fit within a reasonable dimension. The x and y coordinates are evenly spaced points corresponding to retention time 1 and retention time 2.

**Figure 2:**
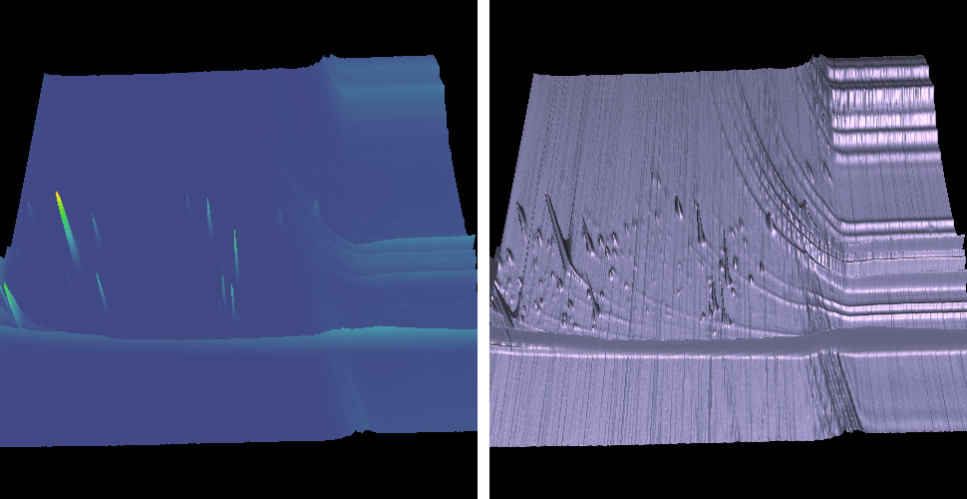
**A color Mapped Height Field**. A color mapped height field (left). Using the high contrast option for height field rendering (right), small peaks and background noise, which might otherwise be hidden, are easily seen.

When used in conjunction with a normal color mapping, this does not actually convey any more information than a two-dimensional TIC image (Figure 2, left). However, this is still a useful technique as data can often be portrayed more effectively by mapping the data values to multiple display parameters, in this case color and height. Not only do the two parameters serve to reinforce each other, but one may overcome deficiencies in the other.

Additionally, as a new application, the height field can be used with alternative color mapping schemes, similar to work done by Linsen et al. with LC-MS data [12]. In this case, the height of the peaks is a useful method for communicating peak intensity compared with the color mapped attribute. As an alternative to color mapping, we also provide a 'high contrast' rendering option for the height field. For this technique we enable OpenGL lighting and create a single light source positioned along the positive z-axis with ambient, diffuse, and specular components. We apply diffuse, specular, and shininess material properties to the polygons. Vertex normals are calculated at each vertex in the mesh, corresponding to each RT1/RT2 coordinate. The end result is a high-gloss, metallic looking rendering with high contrast. With this technique, even small peaks are highlighted and readily noticeable, as seen in Figure 2 (right). Background noise is also highly visible in this view, as it produces a large number of small peaks and valleys.

### TIC color maps

The total ion count visualizations both support color mapping based on intensity, difference, and standard deviations away from a mean. Each of these methods can be configured to use either a continuous or discrete color scheme. For the continuous color scheme, the system uses a set of three curves that allow independent control of the hue, saturation, and brightness.

For the discrete color scheme, the system presents the user with a histogram that displays bin colors and data distribution. The user can modify the number of bins, data range for the bins, and bin colors interactively. The color for each bin can be specified by the user, or the system can automatically generate a color mapping. In each case, values are initially mapped to a logarithmic scale where a larger color range is used to represent small intensities, and the scale of large peaks is greatly reduced.

We provide three different types of visualization modes for the TIC color maps: intensity, difference and standard deviation.

#### Intensity

**Figure 3:**
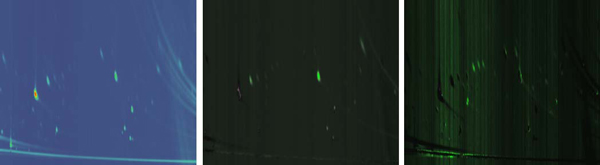
**Color mapping examples**. Examples of the different color mappings that can be applied to total ion count images. Intensity (left), difference (middle), and standard deviations (right).

The intensity mode is a simple mapping of the peak intensity to color. This technique is useful for providing a high-level view of the data. It reveals the location and relative intensity of peaks, and can be useful in helping a user identify any samples that may contain data collection errors. An example of the intensity mode mapping is shown in Figure 3 (left).

#### Difference

The difference mode calculates the difference between two samples. The result is then displayed by simply mapping the difference to a color. The system uses a separate set of HSV curves for positive and negative differences. By default, the hue for negative differences is set to pure green, and the hue for positive differences is set to pure red. An example of the difference mode is shown in Figure 3 (middle). Here, the user can quickly find areas of high positive or negative differences between two samples.

#### Standard deviation

The standard deviation mode also calculates the difference between samples and renders an image based on that difference. However, a color mapping based solely on the magnitude of the difference in intensities may not be what is most interesting. Even using the mean of two sets of samples, the difference in intensity between two large peaks could be relatively high in magnitude compared to two smaller peaks, but this does not necessarily mean that difference is meaningful. By analyzing the standard deviation within user specified groups of samples, differences can be visualized in more certain terms. We create a standard deviation color mapping from a sample group by first calculating a mean TIC for all the samples within that group. Note that the samples are chosen by the user such that they have been pre-normalized as input to the system.

Next a standard deviation TIC is calculated as:

(1) $$\sigma_{A}=\sqrt{\frac{1}{n_{A}}\overset{n}{\underset{i=1}{\sum }}{\left(x_{i}-\mu_{A}\right)}^{2}}$$

Here, *n_A _*is the number of samples in group A, *μ_A _*is the mean TIC of group A, and the *x_i _*are the TICs of the *i*th sample. Once we have computed the standard deviation TIC, it is stored to use for color mapping. This color mapping can then be applied to a sample visualization. Generally, it would be applied to a sample that is part of another group. In order to determine the color at a particular retention time coordinate, the system calculates the corresponding z-value for each point *b *in the new sample, as shown in Equation 2. The z-value is simply how many standard deviations different a value is than the calculated mean. We then use that difference to determine the appropriate color.

(2) $$z=\frac{\mu_{A}-b}{\sigma_{A}}$$

This can help a user to determine whether an observed difference is truly meaningful. Additionally, this technique may effectively reveal areas of difference in smaller peaks that are significant in terms of standard deviations, but were not previously noticed simply because the peaks themselves are smaller. An example is shown in Figure 3 (right), note the green streaks that are not seen in Figure 3 (middle). This helps the user explore regions in the image that are statistically different in a sample when compared to a group of samples.

### Mass spectrum visualization

**Figure 4:**
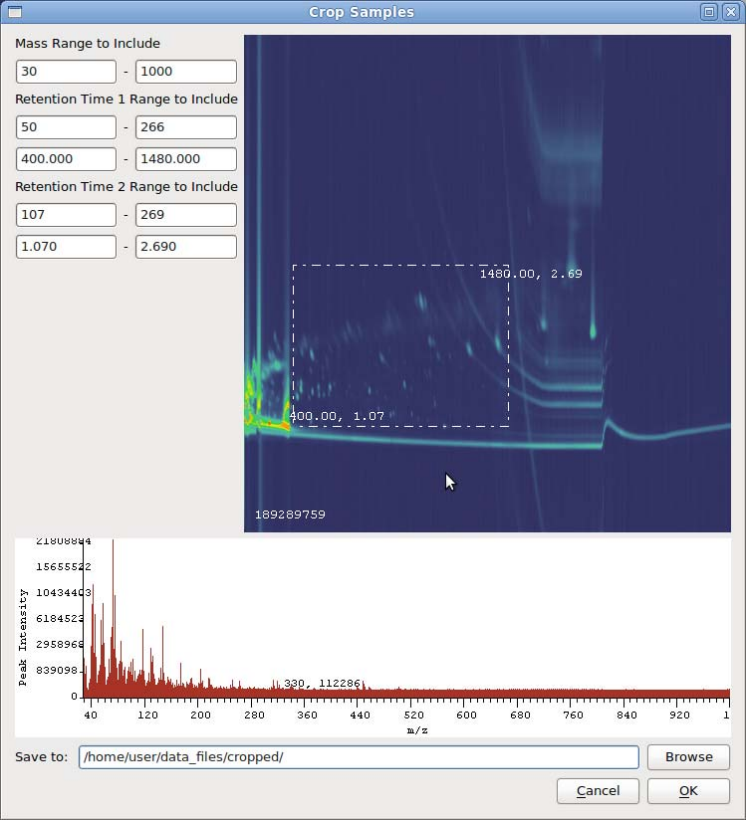
**The OmicsVis tool**. OmicsVis provides users with the ability to crop data sets, enabling them to remove solvent-saturated areas and reduce file size for ease of use.

**Figure 5:**
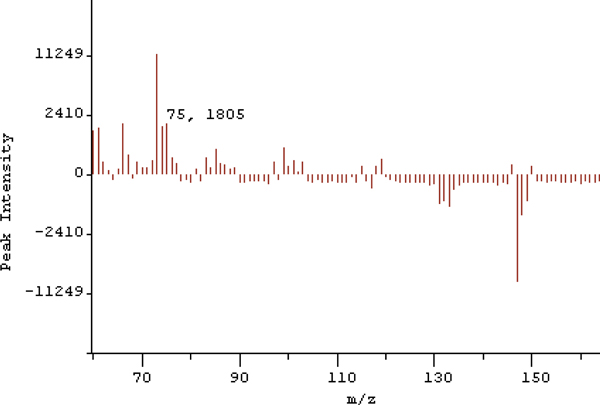
**The Difference Spectrum**. Visualizing the mass spectral difference between two samples.

A mass spectrum view is simply a plot of intensity on the y-axis vs. the mass-to-charge ratio on the x-axis, as seen in Figure 4 (bottom). The user is given the option to plot this spectrum as a bar graph, or as a connected line. In order to help a user form a hypothesis about particular mass values that constitute an observed difference, this system provides a novel technique for visualizing the mass spectral difference between two samples. The technique is similar to rendering a normal mass spectrum. In this case, the zero intensity baseline is drawn across the middle of the viewing window, with bars for positive differences rising upward, and bars for negative differences falling downward from the baseline as demonstrated in Figure 5.

#### Cropping

Datasets created by GCxGC-MS can quickly become extremely large. A typical experiment setup for datasets used in this thesis involve collecting data over a time duration of 40 minutes or more, taking one reading every 10 milliseconds, with each reading containing an intensity value for 900 or more m/z values. Such a setup creates over 850MB of raw data per sample, with experiments containing from 10 to 40 samples. Cropping the datasets not only makes them faster and easier to store and work with, but also allows uninteresting or problematic areas to be removed. Typically, a large amount of solvent will elute near the beginning of the data collection. The high intensities and large variation in this area can skew the scaling and data mapping schemes employed in analysis algorithms. This area can be graphically selected, and cropping can be performed across a group of samples based on those parameters. Figure 4 depicts the use of the cropping tool.

#### Mass filter

When a user is looking for bio-markers and meaningful differences between samples, the user will often identify a few mass values that make up some interesting compound. The mass filter tool allows users to select a unique mass or set of masses from the mass spectrum display. Total ion count data for active TIC visualizations is then re-acquired using only the selected masses. The color mapping is then rescaled to match the new range of values across all of the TIC displays, and the TIC displays are refreshed. This allows a user to quickly determine differences among samples for a selected set of mass values. While other applications provide mass filtering via dialog options, none currently support real-time updating of the mass filter in an interactive manner. Mass values can quickly be added to or removed from the filter as the data sets are being interactively explored for differences in a unique set of mass values.

### Volume visualization

**Figure 6:**
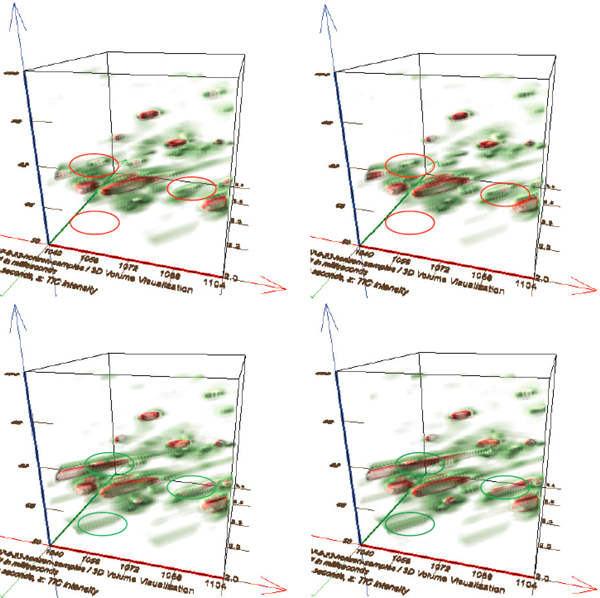
**Volume visualization**. Volume visualization of selected regions (the first retention time: [1104]; the second retention time: [2.0,2.64]; the m/z values: [50,114]). Top: two healthy samples; bottom: two cancer samples. Visible differences favor locating interesting points for further study. Three circles (red for healthy samples and green for cancer samples) indicate three regions with significant differences, whose locations can be easily.

Volume rendering is a well-established method for depicting the embedded structures in a three-dimensional scalar field. The most interesting feature of volume rendering is that important regions can be enhanced while distracting details can be hidden by adjusting their transparency. By incorporating non-photorealistic rendering into volume visualization, volume illustration [13] has proven to be very effective in feature-oriented visualization. The exploration of the mass spectral data can benefit from applying volume visualization in three ways. First, visualizing one dataset with the assistance of a multi-dimensional transfer function gives the user a global picture on the intensity distribution and possible peak patterns. Second, the user can freely select subregions to investigate the local mass spectra. The determination is either interactively specified, or automatically found by comparing the mass spectra in a small-sized window with the mass spectra of known metabolites stored in a database. It also facilitates comparing the spectra of different potential bio-markers. Figure 6 compares a selected region of four samples. Third, by globally or locally comparing the mass spectra of a set of sample data with volume visualization techniques, the user's attention can be quickly directed towards the most interesting features, easing the task of finding or verifying the differences among samples

For datasets whose classifications are known, we can use volume visualization to depict the difference among two classes. For the healthy sample set and the cancer sample set, we compute the mean and normalized variance of the values in each voxel, yielding a template data for each set, respectively. We then visualize the mean values of each template. The variance is used to modulate the opacity after the volume illumination is performed:

(3) $$\alpha_{out}=\alpha_{in}\times \left(1.0-k\times variance\right)$$

where k is an adjustable constant. In our system, k is set to be 0.5. The variance-dependent opacity modulation enables an emphasis of the common features in each class. Thus, color mapping schemes as described in the TIC Color Map section may be applied.

### Mass spectrum based visual exploration

To accurately study the behaviour of a bio-marker or to differentiate different samples, cancer researchers need to check the mass spectrum at given locations, or of a specific potential bio-marker. However, the cycle of statistical bio-marker detection typically produces more than 6,000 candidates. Numerically checking their mass spectra would be a tedious and error-prone procedure. Our novel exploration scheme allows the users to study the mass spectrum of a set of peaks visually with both the two-dimensional and three-dimensional visual representations of the mass spectra. We also provide a comparative visualization approach that is capable of rapidly locating significant bio-marker candidates from a large set of metabolites, thus greatly reducing the user's time, and improving the exploration accuracy.

### Two-dimensional mass spectrum exploration of mouse cancer

The results shown in this section were obtained using raw data exported from LECO's ChromaTOF software. Pre-processing could be applied to remove background levels, normalize samples, and align peaks. In these examples, however, it was interesting to see how the system performs without applying any of these transformations.

This set of samples consists of twenty-nine samples obtained from mice. Ten each were extracted from the body and yolk, while nine were extracted from the head. In each case, half of the samples were exposed to alcohol and half were not. Because alcohol is a depressant and slows down metabolic processes, it is expected that differences between the two samples should show up in the metabolomics data.

The first step taken is to convert the cdf files into raw data files. Since these particular files have very high background levels, the file size drops from about 1.3GB down to 677MB. Compressing the files further reduced the size down to about 245MB. Finally, the samples were cropped to remove regions that were empty or oversaturated with solvent. It was also shown that background level alone occupies mass values above 600, so this area was cropped. The resulting files averaged about 60MB, which can be read from networked file servers in a reasonable amount of time.

**Figure 7:**
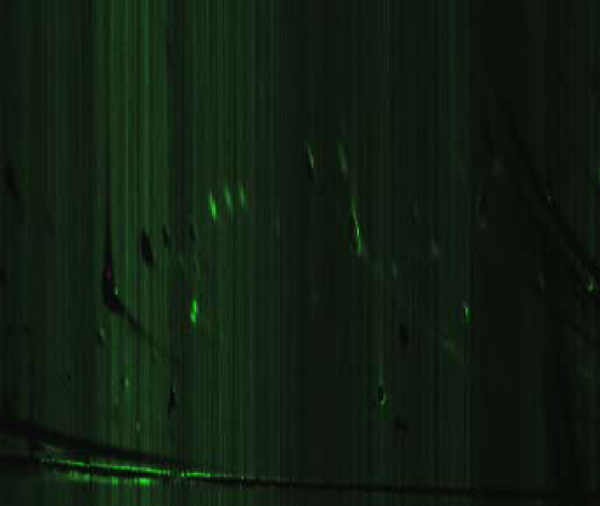
**Alcohol mean colorized by comparing with the mean and standard deviation of the control samples**. The red area indicates an unusual concentration in the alcohol samples.

The first step after converting the data sets is to view the total ion count image from each one. Since each is displayed using a global color scale, quick visual comparison can identify unusual data samples. At this point, one is basically trying to obtain an overview of the data, and look for obvious outliers. For each pair of alcohol and control means, a difference volume was calculated. Mean/standard deviation color mappings were created to help visualize the samples. There is a single red peak that indicates an area of higher concentration in the alcohol samples, but several green peaks can be noticed, standing out against the green hued background that is caused by the higher background level present in the control samples. These areas are also indicated by the mean/standard deviation color mapping in Figure 7. A researcher searching for bio-markers could then target these areas specifically for further analysis.

**Figure 8:**
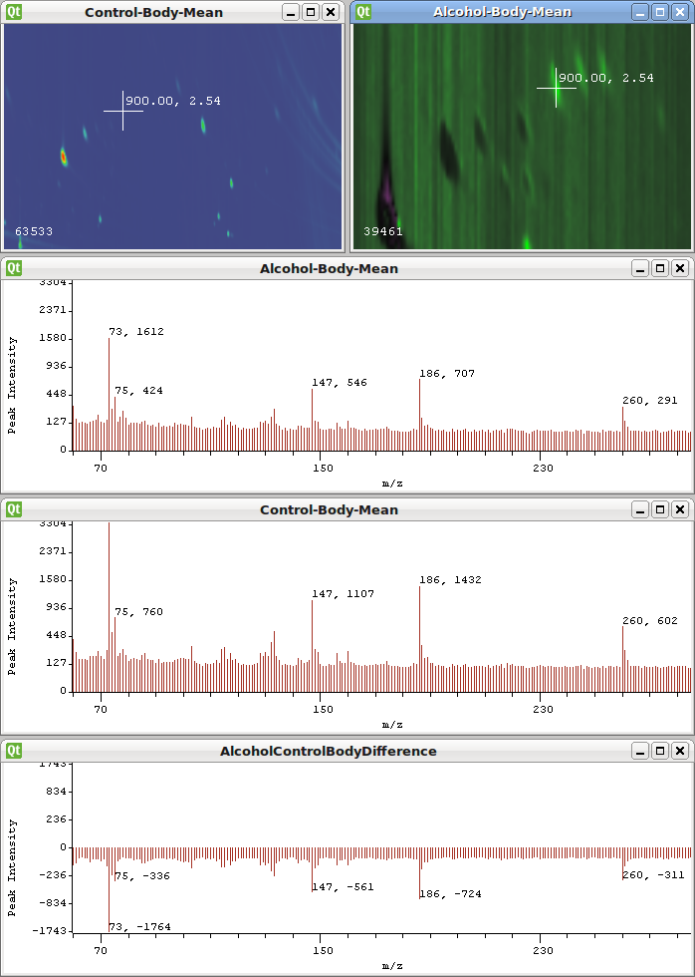
Linked views allow the mass spectra to be explored in real-time as the user interacts with the TIC image.

The linked views included in the system allow a user to investigate the mass spectra in real time. This form of focus+context can be used to form hypothesis about what masses or compounds are contributing the observed differences. Figure 8 demonstrates this feature. Without spending too much time working with these samples, a quick effort can be made using this system to look for meaningful differences between the groups. Using visual inspection, samples with missing or blurry peaks can be eliminated. Averages for the remaining samples in each group can then be computed. From the healthy group,multiple samples can be chosen and used for computation of the mean. While the variations present in these samples make conclusions hard to draw, researchers found such visual exploration techniques to be valuable. Particularly, researchers were able to visually inspect data and rule it out quickly, rather than spending days of analysis time trying to detect features and differences that may be completely obscured.

### Three-dimensional mass spectrum exploration

**Figure 9:**
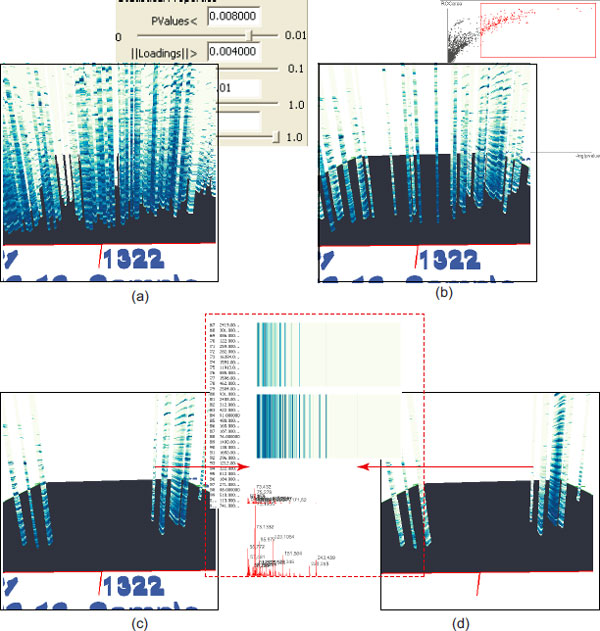
**Exploring differences in canine samples**. Interactive exploration of the 3500 detected peaks in two samples. The user interactively filters the bio-markers by adjusting parameters, (a) is the result (1300 peaks) by changing the thresholds from (0.01, 0.001) to (0.008,0.004) respectively (see the left top snapshot). (b) is the result (198 peaks) by interactively specifying a region in the region operating characteristics plane (see the left top snapshot). (c-d) Comparative exploration of 198 peaks in two samples. For instance, the bio-markers indicated with red arrows exhibit different mass spectra, which can be inspected by comparing their numerical values, color map views, and plot views provided in our system (see the views in the dashed red rectangle).

We can also explore data in a three-dimensional visual representation to depict its properties such as the location, the uncertainty and the weight. This representation uses the (RT1, RT2, MS) triple coordinate space, and is modeled with a cylinder whose height and radius are proportional to the weight and uncertainty respectively, and whose appearance is encoded as a color-mapped texture representing its mass spectrum, as seen in Figure 9. The visual pattern of each peak is fixed during the exploration. The user can freely navigate in this three-dimensional space, visually check the mass spectrum of specific bio-marker candidates, and locate, explore and compare the properties of the selected metabolites of interest.

Our visualization technique compactly displays the mass spectra in a three-dimensional space, thereby easing the understanding and comparison. Although our mode may introduce occlusion problems, a user can annotate significantly different peaks, and employs other provided operations including two-dimensional and three-dimensional spectrum comparisons and statistics-based filtering to quickly reduce the peak number and remove possible visual clutter. The user can progressively filter the bio-marker candidates and the comparison of multiple mass spectra is visually recognizable, and dramatically eases their differentiation. In addition, the user can further search the most significant ones with the assistance of the two-dimensional comparative exploration described below. Figure 9 depicts a simple procedure that locates an interesting potential biomarker (the second metabolite from the left as indicated with an arrow in (c) and (d)).

## Discussion

Initial feedback from researchers working with GCxGC-MS data has been very enthusiastic. This system has provided them with their first opportunity to visualize multiple samples simultaneously. Enthusiasm has also been expressed about mean, standard deviation, and difference calculation for a set of samples. By visualizing these calculated data, the human eye can quickly identify differences. This system can be used to identify a particular peak or region of difference, and then the mass spectra can be explored to provide validation of differences, and hypotheses about the compounds involved. With this information, their existing tools can be used to obtain information about compound identification and intensity details much more quickly than was previously possible.

The researchers also frequently mentioned how pleased they were with the speed of the software. Other commercial systems will often take tens of seconds or minutes to even display a TIC image. Additionally, these software systems allow masses to be filtered, and individual spectra to be visualized, however, it is a slow and cumbersome process to change parameters and redisplay a new spectra or filter out different mass values. No other system currently used by this group was able to provide the fast, interactive filtering and mass spectra exploration of our system. As an example, the researchers can now quickly change the mass filter for a specific value, and slowly move through the entire range of mass values. Multiple samples can be visualized, and as the mass filter is updated the researchers can very quickly visually identify cases in which a unique mass has an unusual abundance in some samples. Currently, a version of this system is deployed for use on the Cancer Care Engineering Hub at Purdue University, http://ccehub.org.

The volume visualization tools have also proven to be effective for comparing the selected region of different samples. For instance, Figure 6 reveals some differences between the samples with and without breast cancer in the bottom left portion. Based on the visualization results, the researchers were able to locate the position and check the detailed mass spectrum with the three-dimensional representation. However, we have found that visualizing a large-sized dataset is likely too cluttered to find significant differences easily. In the future, we expect to explore more powerful data visualization toolkits such as spectra-dependent transfer functions to address this problem. These approaches can easily be incorporated into the present visualization framework.

## Conclusions and future work

Our system provides several benefits to researchers. We have found that the ability to visualize multiple samples and explore the data interactively greatly adds to one's understanding, particularly for users who were previously unfamiliar with the data. Sample visualization allows researchers to quickly validate some level of quality and consistency in their data. Using visual analysis and exploration can help researchers identify differences and bio-markers more quickly than the traditional, purely analytic approaches. Finally, we have found that being able to visually analyze the data increases the level of confidence in the results obtained.

As our system was evaluated, we also received several suggestion for future work. Plans for future work involve producing output that can be used by other tools. For example, we could allow the user to select a single peak (or what appears to be a single peak) from a TIC image. This could be used to reconstruct a one-dimensional chromatogram for that region, and input that data into other existing tools that would then perform peak deconvolution (if necessary) and identification. Finally, many of these features could benefit from incorporating gradient based value mapping into the display. This was applied to GCxGC datasets in [14], and could be very effective when used with the types of comparative visualization techniques provided by this system.

## List of abbreviations used

GC × GC-MS: Gas chromatography × Gas Chromatography - Mass Spectrometry Data; RT1: Retention Time 1; RT2: Retention Time 2; TIC: Total Ion Count; LCMS: Liquid Chromatography Mass Spectrometry; OPLS: Orthogonal Partial Least Squares.

## Competing interests

The authors declare that they have no competing interests.

## Authors' contributions

PL carried out the system design and implementation and helped draft the manuscript. RM carried out the system design, provided input on the implementation, coordinated the project and helped draft the manuscript. WC carried out the system design and implementation. DSE participated in the design of the project and helped to draft the manuscript. All authors read and approved the final manuscript.
